# High-efficiency dye-sensitized solar cells based on robust and both-end-open TiO_2 _nanotube membranes

**DOI:** 10.1186/1556-276X-6-475

**Published:** 2011-07-27

**Authors:** Jia Lin, Jingfei Chen, Xianfeng Chen

**Affiliations:** 1 Department of Physics, The State Key Laboratory on Fiber Optic Local Area Communication Networks and Advanced Optical Communication Systems, Shanghai Jiao Tong University, 800 Dongchuan Road, Shanghai 200240, China

## Abstract

In the present work, dye-sensitized solar cells (DSSCs) were fabricated by incorporating transparent electrodes of ordered free-standing TiO_2 _nanotube (TNT) arrays with both ends open transferred onto fluorine-doped tin oxide (FTO) conductive glass. The high-quality TiO_2 _membranes used here were obtained by a self-detaching technique, with the superiorities of facile but reliable procedures. Afterwards, these TNT membranes can be easily transferred to FTO glass substrates by TiO_2 _nanoparticle paste without any crack. Compared with those DSSCs consisting of the bottom-closed membranes or attached to Ti substrate, the carefully assembled and front-side illuminated DSSCs showed an enhanced solar energy conversion efficiency as high as 5.32% of 24-μm-thick TiO_2 _nanotube membranes without further treatments. These results reveal that by facilitating high-quality membrane synthesis, this kind of DSSCs assembly with optimized tube configuration can have a fascinating future.

## Introduction

The application of semiconductor TiO_2 _in dye-sensitized solar cells (DSSCs) was extensively investigated for its low cost and high energy conversion efficiency. The high-efficiency DSSCs were first reported in 1991 by O'Regan and Grätzel with a power conversion efficiency of 7.12% [1]. The photoanodes are consisted of disordered TiO_2 _anatase nanoparticle films, with sufficient dye anchored for high light harvesting, on transparent conducting oxide glass. The latest certified efficiency of DSSCs, which are based on the nanoparticulate TiO_2 _photoanode, is 11.2% [2]. However, the losses in nanoparticulate DSSCs were large because of the carrier recombination at grain boundaries and long carrier diffusion paths through the TiO_2 _nanoparticle network [3]. Compared with nanoparticles, highly ordered vertically oriented TiO_2 _nanotube (TNT) arrays cannot only offer a large internal surface area but also reduce recombination probabilities and provide a directed electron traveling path [4]. DSSCs with pure TiO_2 _nanotubes [5-9] and treated TiO_2 _nanotubes [10,11] (treated with TiCl_4 _for instance) both show good performance on photon-to-current conversion efficiency. The best efficiency records are 5.2% and 7%, respectively [3].

DSSC with the photoanode of TNTs grown on Ti foil requires backside illumination, which may cause light reflection and absorption by the counter electrode and the electrolyte [12,13]. To resolve this drawback, Grimes and co-workers demonstrated a transparent TiO_2 _nanotube-based photoanode by an anodization of Ti thin film sputtered on fluorine-doped tin oxide (FTO) conductive glass [14]. However, procedures for photoanode fabrication were complex and costly, which required special treatment of the metal layer in contact with the electrolyte surface [15] and strict process control [16]. In addition, the increase in film thickness will lead to the poor adhesion to the substrate [17-19]. Free-standing nanotubes detached from Ti substrate and fixed onto the FTO glass is another approach to prepare photoanode of front-side illuminated DSSCs. Chen and Xu developed a two-step anodization process to fabricate large-area free-standing TiO_2 _nanotube arrays and transferred them onto FTO glass [20]. Lei et al. reported the formation of large-scale free-standing TNT arrays via sonication of TNT arrays on Ti foil and transferred them onto the FTO glass [21]. In these cases, the bottom ends of the nanotubes are closed. Recently, Lin et al. introduced a transparent photoanode made of ordered opened-end TNT arrays transferred onto FTO glass and observed an increase in efficiency than closed-end TNTs [22]. However, it needed an additional chemical etching step to open the closed bottom end, and due to the complex fabrication procedures, this cell configuration has not been paid extended attention.

Recently, we reported a facile fabrication rote to synthesis free-standing TiO_2 _nanotube membranes, with both the closed and open bottom-side morphologies, through the so-called self-detaching anodization [23]. The films with high-quality surfaces and both-side-open tubes could be obtained by appropriate thermal treatment during the process. This self-detaching process is easy and efficient without additional chemical dissolution or etching. So we expect that by facilitating the synthesis process and improving film quality, high-quality DSSCs based on both-end-open tubes can have broad prospects. Herein, we report our results on application of these transparent, free-standing TNT membranes for use on the photoanodes in DSSCs. Photoanodes consisting of high-quality TiO_2 _membranes well attached to the FTO can be successfully fabricated. Under air mass (AM) 1.5 G solar light, the DSSC based on the 24-μm-thick tubes without TiCl_4 _treatment and under not-optimized conditions shows the best solar cell efficiency of 5.32%, which is consistent with the current recorded efficiencies. By further optimizing the tube length, annealing temperature and electrolyte composition, the efficiency can be expected to be further improved.

## Experimental

A detailed methodology of fabricating free-standing TNT membranes via self-detaching anodization has been mentioned elsewhere [23]. Hence, we only summarized the key points of the fabrication processes here. To grow TNT arrays on Ti, a common two-electrode electrochemical cell was used with the working electrode Ti foil (0.2 mm thickness, Strem Chemicals, Newburyport, MA, USA) and the counter electrode Pt foil. Anodization was carried out in an ethylene glycol electrolyte containing 0.5 wt.% NH_4_F + 3 vol.% deionized water under a constant voltage of 60 V. The second-step anodization durations are 0.5 and 1 h for different film thicknesses, and the resulting oxide films were subjected to thermal treatments at 200°C and 400°C for both-end-opened and bottom-end-closed membranes, respectively. Afterwards, the as-formed films were completely detached by the third-step anodization in about 1 h under a temperature of 30°C.

A TiO_2 _nanoparticle (TNP) viscous paste was prepared as follows: mixed TiO_2 _nanoparticles (P25, Degussa, Borger, TX, USA) with 3 vol.% acetic acid solution at the weight rate of 3:10 and stirred for 1 h. The TNP paste was spin-coated onto FTO glass substrates, and the free-standing TiO_2 _nanotube membranes were transferred onto the paste layers immediately. After being dried in air, the films were sintered at 450°C for 3 h. The as-formed electrodes were then immersed in a 1:1 (*v*/*v*) acetonitrile and ethanol solution containing 3 × 10^'4 ^M RuL_2_(NCS)_2_:2TBA (N719 dye, L = 2,2'-bipyridyl-4,4'-dicarboxylic acid, TBA = tetrabutylammonium, Dyesol, Queanbeyan, New South Wales, Australia) for 24 h. The sensitized electrodes were further sandwiched with the sputtered-Pt FTO glass, separated by a 60-μm-thick hot-melt spacer. The intervening space was filled with a common kind of liquid electrolyte of DMPII/LiI/I_2_/TBP/GuSCN in acetonitrile (DHS-E23, Heptachroma, Dalian, China).

Four kinds of DSSCs were prepared for investigation: first, photoanode made of free-standing TNT membrane with both ends open transferred onto FTO glass (O-FTO); second, photoanode made of free-standing TNT membrane with the bottom ends closed transferred onto FTO glass (C-FTO); third, photoanode made of TNP layer (approximately 10 μm) pasted on FTO by doctor blade technique (NP-FTO); and fourth, photoanode made of TNT arrays on Ti substrate (NT-Ti), with a film thickness of about 24 μm. The third and fourth samples are for photoelectric performance comparison. All the TNTs in our experiments are pure without any treatment.

Field emission scanning electron microscope (FE-SEM, FEI Sirion 200, FEI Company, Hillsboro, OR, USA) was utilized for morphological and structural characterization. Photocurrent-voltage characteristics (*J*-*V *curves) were measured under AM 1.5 G solar simulator (Oriel Sol3A, Newport Corporation, Irvine, CA, USA) at a light intensity of 100 mW/cm^2^.

## Results and discussion

**Figure 1:**
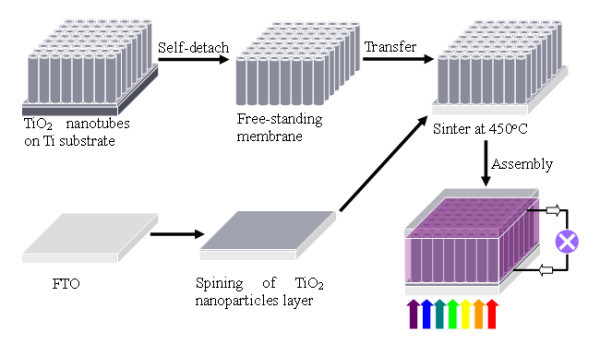
**Scheme of a front-side illuminated DSSC fabrication**.

Using the self-detaching method, free-standing membranes were prepared. Then, the membrane was strongly adhered onto FTO glass by TiO_2 _nanoparticle (TNP) paste and sintered at 450°C. Afterwards, the photoanode was subjected to an assembly process to fabricate DSSC. The scheme of a front-side illuminated DSSC fabrication is shown in Figure 1.

**Figure 2:**
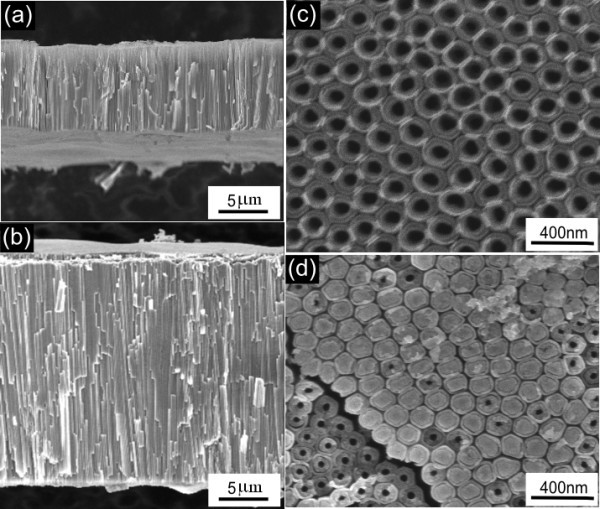
**FE-SEM images of self-detached free-standing TNT membranes**. Cross-section view of TNT membranes with (**a**) 0.5 h and (**b**) 1 h of second-step anodization; the bottom view of membrane subjected to the thermal treatment at (**c**) 200°C and (**d**) 400°C.

Figure 2 gives the FE-SEM images of self-detached free-standing TNT membranes. Figure 2a, b shows the cross sections of TNT membranes whose durations of second-step anodization were 0.5 and 1 h, respectively. A tube length of 9.72 μm can be obtained in 0.5 h and 23.8 μm in 1 h. Figure 2c shows the bottom view of membrane subjected to the thermal treatment at 200°C. It can be seen that highly ordered TiO_2 _nanotube arrays are close-packed together with all the bottom ends open. Figure 2d shows the bottom view of membrane subjected to the thermal treatment at 400°C. Almost all the tube ends are closed.

**Figure 3:**
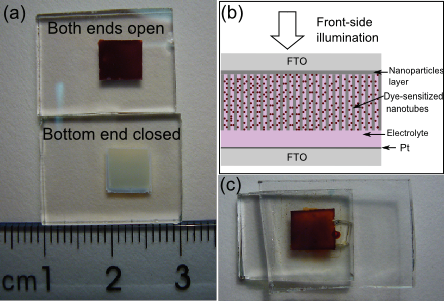
**Optical images and schematic illustration for the preparation of front-side illuminated DSSCs**. **(a) **Optical image of the free-standing membranes adhered on FTO glass. (**b**) Schematic illustration of DSSC fabricated with free-standing TNT membrane. (**c**) Optical image of our DSSC sample with the front side upturned.

As discussed above, the adhesion quality of the membranes to the FTO substrate is quite satisfied. Figure 3a presents the optical image of the free-standing membranes adhered on FTO glass. The upper one is the membrane with both ends open. This membrane is amorphous and not optically transparent. After being annealed at 450°C, the membrane will become transparent. The lower sample in Figure 3a is the membrane with the bottom end closed. This membrane has been thermal treated at 400°C during the detaching process and crystallized in the anatase phase. After being annealed at 450°C, the samples were sensitized by N719 dye solution and assembled into DSSCs. The schematic illustration of DSSC fabricated with free-standing TNT membrane is shown in Figure 3b. Figure 3c is the optical image of our DSSC sample with the front side upturned. There is a small piece of glass pasted on the backside to cover the pore for electrolyte injection.

**Figure 4:**
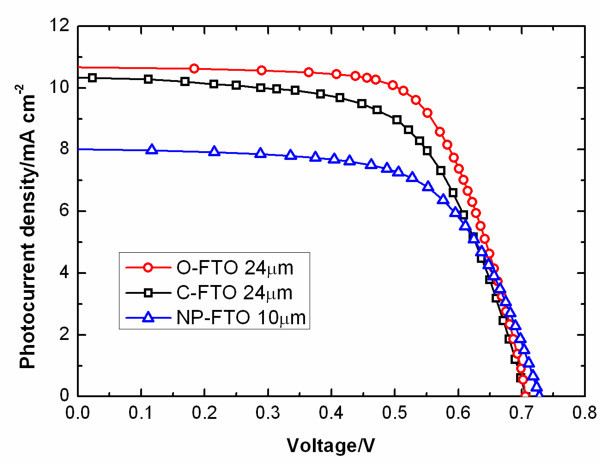
***J*-*V *curves of DSSCs based on 24-μm O-FTO, 24-μm C-FTO, and 10-μm NP-FTO**.

The photocurrent-photovoltage (*J*-*V*) properties of DSSCs based on 24-μm O-FTO and 24-μm C-FTO as well as a 10-μm NP-FTO measured under AM 1.5 G illumination (100 mW/cm^2^) are displayed in Figure 4. For comparison, the NP-FTO-based DSSC exhibited a short-circuit photocurrent density (*J*_sc_) of 8.04 mA/cm^2^, open circuit voltage (*V*_oc_) of 0.73V, fill factor (FF) of 0.64, and efficiency (*η*) of 3.75%. The C-FTO-based DSSC shows improved performance, with *J*_sc _of 10.32 mA/cm^2^, *V*_oc _of 0.71V, FF of 0.62, and *η *of 4.52%. Efficiency increased by 20% compared to NP-FTO-based DSSC. With O-FTO as the photoanode, *J*_sc _of the DSSC increased to 10.65 mA/cm^2^, with *V*_oc _of 0.70 V, FF of 0.70, and *η *of 5.32%. The fill factor also increased with the elimination of the barrier layer which reduced the series resistance [24]. Efficiency increased by 18% compared to C-FTO-based DSSC. The lower efficiency of C-FTO-based DSSCs was probably attributed to the presence of the barrier layer existed at the bottom side which may hinder solar light, active material, and electron transport and diffusion.

**Figure 5:**
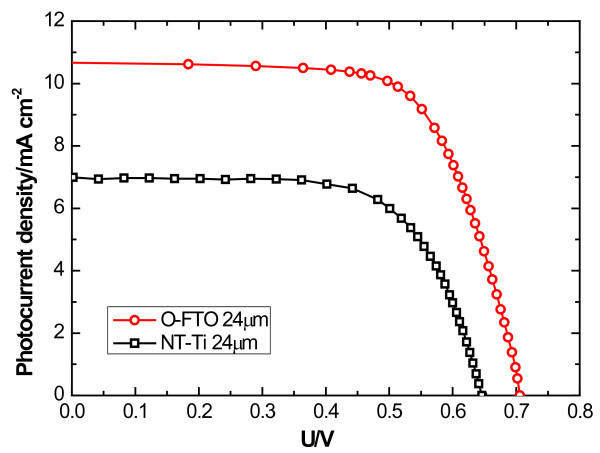
***J*-*V *curves of DSSCs based on O-FTO and NT-Ti with the same film thickness**. Thickness is 24 μm.

Figure 5 shows the *J*-*V *curves of DSSCs based on O-FTO and NT-Ti with the same film thickness (approximately 24 μm) measured under AM 1.5 G illumination (100 mW/cm^2^). The O-FTO-based DSSC was front-side illuminated, and the NT-Ti-based one was backside illuminated. It clearly shows that the *J*_sc _(6.99 mA/cm^2^) and *V*_oc _(0.64V) of the back-illumination mode are significantly smaller than that of the front illumination mode (10.65 mA/cm^2 ^and 0.70 V). The efficiency of O-FTO-based DSSC is 5.32%, which is much higher than the NT-Ti one of 3.04%. That is possibly because the counter electrode (Pt) and the electrolyte (I¯/I_3_¯) attenuate the incident light intensity for the backside illumination mode, and also, a rutile phase was easy to be formed at the interface between the barrier layer and Ti substrate when annealed at high temperature, which may have a bad effect on charge collection [24].

**Figure 6:**
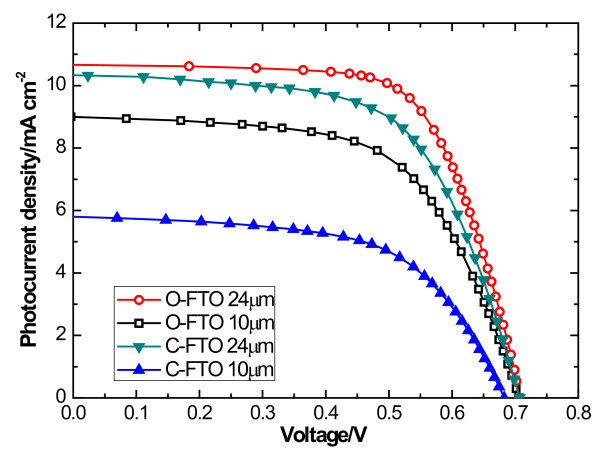
***J*-*V *curves of DSSCs based on O-FTO and C-FTO with different film thicknesses**.

Of the same kind of photoanode, longer nanotube length makes higher photoelectric performance. Figure 6 is the *J*-*V *curves of DSSCs based on O-FTO and C-FTO with different film thicknesses measured under AM 1.5 G illumination (100 mW/cm^2^). This can be possibly attributed to the increase in the number of absorbed dye molecules from the increased surface area of the nanotube membranes. Therefore, the light-harvesting efficiency was improved and photogenerated electron density was also increased.

Table 1 summarizes the values of *J*_sc_, *V*_oc_, FF, and *η *for all the DSSC samples. It is clearly shown that the O-FTO-based DSSCs with the film thickness of 24 μm have the best photoelectric performance.

**Table 1:** The values of *J*_sc_, *V*_oc_, FF, and *η *for all the DSSC samples

Photoanode	Thickness (μm)	*J*_sc _(mA/cm^2^)	*V*_oc _(V)	FF	*η *(%)
O-NT/FTO	24	10.65	0.70	0.70	5.32
O-NT/FTO	10	9.00	0.71	0.61	3.86
C-NT/FTO	24	10.32	0.71	0.62	4.52
C-NT/FTO	10	5.82	0.68	0.59	2.35
NP/FTO	10	8.04	0.73	0.64	3.75
NT/Ti	24	6.99	0.64	0.68	3.04

## Conclusions

Front-illuminated DSSCs based on both-end-opened TNT membranes, which were prepared by a simple self-detaching method, were achieved with the photoanodes consisting of these membranes adhered on FTO glass by TNP paste. Compared with NT-Ti-based backside illuminated DSSCs with an efficiency of 3.04% and C-FTO-based DSSCs with an efficiency of 4.52%, the O-FTO-based DSSCs with a film thickness of 24 μm showed the best efficiency of 5.32%. The simple and reliable assembly of this high-efficiency solar cell configuration can open new prospect for future DSSC application.

## Competing interests

The authors declare that they have no competing interests.

## Authors' contributions

XC proposed the idea and presided over the study. JL and JC conceived and designed the experiments. JL and JC wrote the paper. All authors read and approved the final manuscript.
